# Retraction Note: Future of low back pain: unravelling IVD components and MSCs’ potential

**DOI:** 10.1186/s13619-024-00199-6

**Published:** 2024-08-05

**Authors:** Raquel Leão Monteiro

**Affiliations:** https://ror.org/04988re48 grid.410926.8 0000 0001 2191 8636 P. Porto: Instituto Politécnico Do Porto, ESS, Porto, Portugal

**Retraction Note** **:** **Cell Regeneration 13, 1 (2024)**

**https://doi.org/10.1186/s13619-023-00184-5**

The author has retracted this article because they did not have permission to publish the material contained therein. This has been confirmed by an investigation by i3S - Institute for Research and Innovation in Health of the University of Porto. The author agrees with this retraction.

